# Assessment of a screening tool to aid home-based identification of adolescents (aged 10–14) living with HIV in Zambia and South Africa: HPTN 071 (PopART) study

**DOI:** 10.1371/journal.pone.0266573

**Published:** 2024-02-16

**Authors:** Mwate Joseph Chaila, David Mcleod, Sten H. Vermund, Moomba Mbolongwe-Thornicroft, Madalitso Mbewe, Constance Mubekapi-Musadaidzwa, Abigail Harper, Albertus Schaap, Sian Floyd, Graeme Hoddinott, Richard Hayes, Sarah Fidler, Helen Ayles, Kwame Shanaube, on behalf of the HPTN 071 (PopART) for Youth (P-ART-Y) Study Team

**Affiliations:** 1 Zambart, University of Zambia School of Medicine, Ridgeway Campus, Lusaka, Zambia; 2 Department of Infectious Disease Epidemiology, London School of Hygiene and Tropical Medicine, London, United Kingdom; 3 Yale School of Public Health, New Haven, Connecticut, United States of America; 4 Faculty of Medicine and Health Sciences, Desmond Tutu TB Centre, Department of Paediatrics and Child Health, Stellenbosch University, Cape Town, South Africa; 5 Department of Medicine, Imperial College London, London, United Kingdom; International AIDS Vaccine Initiative, UNITED STATES

## Abstract

**Introduction:**

The HPTN071 (PopART) for Youth (P-ART-Y) study evaluated the acceptability and uptake of a community-level combination HIV prevention package including universal testing and treatment (UTT) among young people in Zambia and South Africa. We determined whether a four-question primary care level screening tool, validated for use in clinical settings, could enhance community (door-to-door) identification of undiagnosed HIV-positive younger adolescents (aged 10–14) who are frequently left out of HIV interventions.

**Method:**

Community HIV-care Providers (CHiPs) contacted and consented adolescents in their homes and offered them participation in the PopART intervention. CHiPs used a four question-screening tool, which included: history of hospital admission; recurring skin problems; poor health in last 3 months; and death of at least one parent. A “yes” response to one or more questions was classified as being “at risk” of being HIV-positive. Rapid HIV tests were offered to all children. Data were captured through an electronic data capture device from August 2016 to December 2017. The sensitivity, specificity, positive predictive value and negative predictive value were estimated for the screening tool, using the rapid HIV test result as the gold standard.

**Results:**

In our 14 study sites, 33,710 adolescents aged 10–14 in Zambia and 8,610 in South Africa participated in the study. About 1.3% (427/33,710) and 1.2% (106/8,610) self-reported to be HIV positive. Excluding the self-reported HIV-positive, we classified 11.3% (3,746/33,283) of adolescents in Zambia and 17.5% (1,491/8,504) in South Africa as “at risk”. In Zambia the estimated sensitivity was 35.3% (95% CI 27.3%-44.2%) and estimated specificity was 88.9% (88.5%-89.2%). In South Africa the sensitivity was 72.3% (26.8%-94.9%) and specificity was 82.5% (81.6–83.4%).

**Conclusion:**

The sensitivity of the screening tool in a community setting in Zambia was low, so this tool should not be considered a substitute for universal testing where that is possible. In South Africa the sensitivity was higher, but with a wide confidence interval. Where universal testing is not possible the tool may help direct resources to adolescents more likely to be living with undiagnosed HIV.

**Trial registration:**

**Clinical Trial Number:** NCT01900977.

## Introduction

HIV infection among adolescents remains a challenge, with ≈1.8 million adolescents estimated to be living with HIV globally in 2015 [1]. In Africa, HIV/AIDS is now the leading cause of death among adolescents, notably among girls, and is ranked second globally next to unintentional injuries [1,2]. Furthermore, an estimated 250,000 adolescents aged 15–19 become newly HIV-positive annually [3]. Compared to adults, older adolescents, and children/infants, little is known about the burden of HIV and AIDS among young adolescents (aged 10–14 years) as data on them are rarely collected, and the statistics that are reported aggregate them with older youth. The other challenge is that, in many high burden countries, this age group requires parental consent for the provision of sexual and reproductive health service [1,4,5].

Another reason that very few studies report HIV testing uptake, knowledge levels, and behaviours in 10–14 year olds is that this age group is rarely a target group for HIV testing [6,7]. This has likely disadvantaged programmatic planning for HIV testing, treatment and prevention services for young adolescents [7]. Although estimates of the HIV prevalence in this 10–14-year-old age group were limited, among 15–19-year-olds in Zambia, in 2018, the prevalence was estimated to be 2.6% and 1.2% among females and males respectively [8]. In South Africa in the same age group the estimates were 5.9% in females and 4.1% in males in 2016 [9].

Older children and adolescents infected perinatally often remain undiagnosed with HIV-infection and as such would not receive antiretroviral therapy (ART) [10–13]. Efforts to identify such undiagnosed adolescents have been implemented in sub-Saharan Africa; a systematic review and other studies have shown that while provider-initiated testing and counselling (PITC) will capture some youth, additional interventions at the community level are needed to reach more youth, for example, through family-based testing, index testing with parents or family members as entry points, and distribution of HIV self-testing kits [12,14,15].

In 2011, Ferrand et al. developed a primary care level algorithm (screening tool) for identifying adolescents living with HIV in populations at high risk of vertical transmission. The screening tool consisted of five basic questions to identify adolescents aged 10–19 attending primary care facilities, who were more likely to be at risk of being HIV-positive in Zimbabwe[16]. The questions were further reduced from five to four for use in children and adolescents aged 6–15 years[10]. These four questions were later applied in community settings of Zimbabwe for children and adolescents aged 8–17 years [17].

Using the data collected during the PopART for Youth (P-ART-Y) study, we sought to determine whether this four-question screening tool could efficiently identify undiagnosed adolescents living with HIV (ALHIV) aged 10–14 in community-level interventions in Zambia and South Africa. The design of the PopART intervention, which was conducted through a door-to-door approach, allowed us to address this question using a very large sample of adolescents.

## Methods

### Trial design and setting

The PopART for Youth (P-ART-Y) study aimed to determine the acceptability and uptake of HIV testing among adolescents and young adults aged 10–24 years (young people) and was implemented from October 2015 to December 2017; the main findings from the study have been published elsewhere [13,18,19]. The P-ART-Y study was nested within the HPTN071 (PopART) trial, a three-arm cluster-randomized trial aimed at assessing the impact of a combination HIV prevention package on community-level HIV incidence [13,20].

The design of the PopART trial has been described previously [20], but briefly it was conducted in 12 communities in Zambia and nine communities in South Africa between November 2013 and December 2017. The 21 communities were randomly allocated to one of three arms. In Arm A, a combination HIV prevention package was delivered door-to-door by Community HIV-care Providers (CHiPs) and people living with HIV in arm A had access to antiretroviral therapy (ART) regardless of CD4+ cell count. Members of arm B communities received the same package, but ART was initially provided according to national ART guidelines. Arm C received standard of care services. National guidelines were changed following World Health Organization (WHO) recommendations in 2016 so that all three arms began receiving ART regardless of CD4+ cell count [21].

The intervention was to be offered annually in all 14 intervention communities (Arm A and Arm B) in three annual rounds, although in practice these rounds took longer than expected to deliver and lasted approximately 15 to 20 months. The CHiPs recorded basic data on the household and all household members on an electronic data capture device, as well as more detailed data such as HIV test history and HIV test results of all consenting participants. Details of the PopART intervention, informed consent, and HTS are described elsewhere [13,20,22,23].

### The P-ART-Y study and screening tool

The P-ART-Y study was implemented during the second and third annual rounds of the PopART intervention between October 2015 to December 2017. It was implemented in three phases: the qualitative baseline study and collection of data from the on-going HPTN071 (PopART) trial (phase 1) [20], addition of youth-targeted interventions in communities (phase 2) which included integration of school based intervention in all study communities; and a cross-sectional survey to measure the knowledge of HIV status in Arm C so that it could be compared with Arms A and B to see how the intervention changed knowledge of HIV status (phase 3) [13].

During household visits CHiPs asked four questions to parents, guardians, or other caretakers of adolescents aged 10–14 years. The questions were drawn from a previously developed primary care-level screening algorithm [10], and were asked prior to the offer of an HIV test. A “yes” response to one or more of the following questions meant that the adolescent was considered to have an increased risk of being HIV-positive (“at risk”):

1. Has the child ever been admitted to hospital?
2. Does the child have recurring skin problems?
3. Are one or both parents of the child deceased?
4. Has the child had poor health in the past 3 months?

All adolescents present were offered an HIV test, irrespective of screening result. For adolescents not present CHiPs would return at a later date to offer a test. If an adolescent screened “at risk” greater efforts were made to locate them, with an average of three attempts made to find and test them.

The objective of the analysis reported here was to assess the effectiveness of using this screening tool to identify 10–14-year-olds at greater risk of being HIV-positive. All children aged 10–14 years old who participated in the intervention, did not report to CHiPs being HIV-positive and had a result from a rapid HIV test administered by a CHiP were included in the analysis. This information was only collected in the PopART intervention arms (A and B) and so no data from arm C are included. The data are from the third PopART intervention round (R3), which took place between August 2016 and December 2017.

### Data collection and analysis

The CHiPs recorded all household data in an electronic data capture device. Proportions of adolescents who were deemed “at risk” were calculated and the association of screening “at risk” with age, sex and community was tested using a chi-squared test. In order to check if the screening tool was predictive of HIV status, the association between testing HIV-positive and screening at-risk was estimated using a multilevel logistic regression, including all participants who had a screening result and an HIV test result. This regression included a random effect for CHiPs work zones, as both the exposure (screening result) and the outcome (HIV status) could be clustered by location. The model was also adjusted for the participants’ age, sex and community as fixed effects.

To assess the sensitivity, specificity, and the positive and negative predictive values of the screening tool, both the screening result and the HIV test result (the gold standard) needed to be available, so again this analysis was restricted to those who accepted an HIV test from the CHiPs.

Given that CHiPs prioritised adolescents screening “at-risk” for follow up if they were absent at first visit, this meant that HIV testing uptake was higher in “at-risk” adolescents. To account for this imbalance, the subsequent estimates for sensitivity and specificity were weighted using inverse-probability weights to take into account the fact that the true HIV status was known for a greater proportion of the individuals in the “at risk” group. The weights were the inverse of the proportion who received a test in each of the “at-risk” or “not-at-risk” groups.

It was assumed that acceptance of testing was independent of HIV status among those in the same risk group, i.e., within each risk group the prevalence of HIV was the same among those who accepted testing and those who declined. The prevalence of HIV-positive status among those who were not previously known to be HIV-positive was estimated in each country, again using the inverse probability weights to account for the difference in uptake of testing by risk group. Stata version 16 was used for all data management and analysis.

### Ethical approval

Ethics approval was obtained from the ethics committees of the University of Zambia, Stellenbosch University and the London School of Hygiene and Tropical Medicine. Permission to conduct the study was received from the Zambian Ministry of Health and the Western Cape Department of Health in South Africa. We sought both informed consent from parents/guardians and assent from adolescents [13].

## Results

### Participation

**Fig 1:**
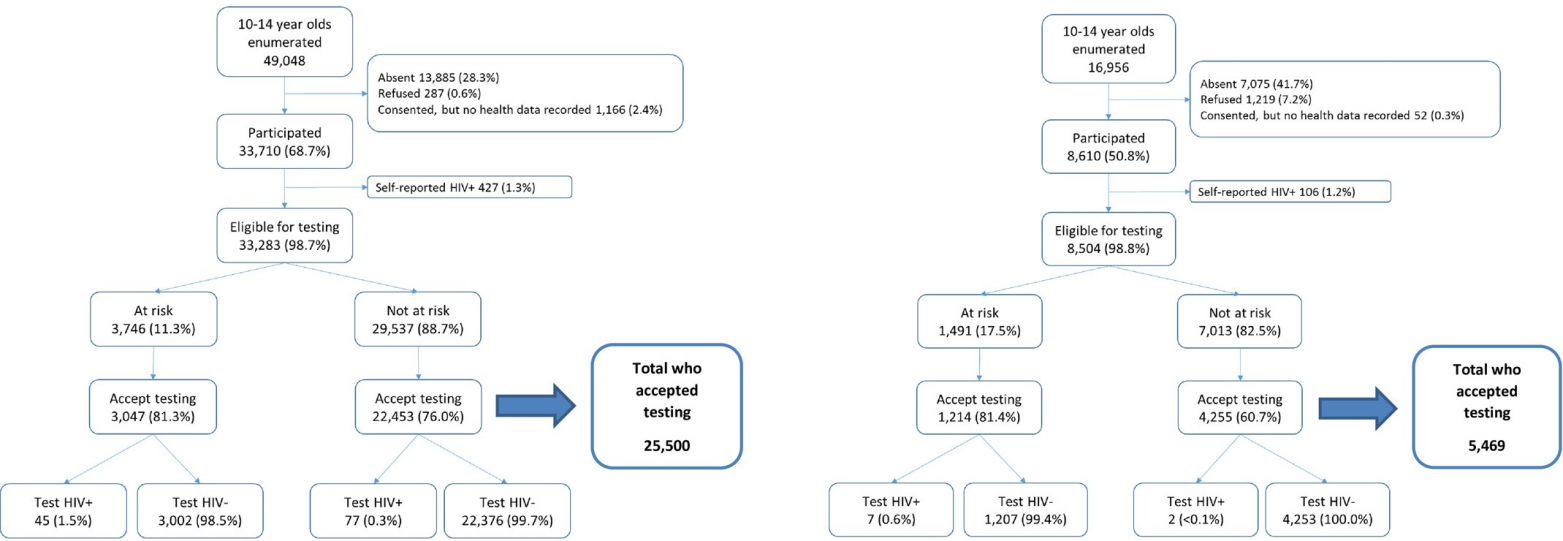
a. Flow chart for the HIV testing cascade in adolescents aged 10–14 years (Zambia) in the P-ART-Y sub-study of HPTN 071 (PopART). b. Flow chart for the HIV testing cascade in adolescents aged 10–14 years (South Africa) in the P-ART-Y sub-study of HPTN 071 (PopART).

There were 49,048 adolescents aged 10–14 enumerated within the eight Zambian communities during R3, of whom 33,710 (68.7%) participated in the PopART intervention. Absence from the household at the time of the CHiPs team visit was the primary reason for non-participation (90.5% of all non-participants). Among these adolescents, 427 (1.3%) self-reported being HIV-positive leaving 33,283 participants eligible for HIV testing by CHiPs (Fig 1A).

In South Africa 16,956 adolescents were enumerated with 8,610 (50.8%) participating in the intervention. Again, most non-participation (84.8%) was due to absence from the household. There were 106 (1.2%) participants who self-reported being HIV-positive, resulting in 8,504 participants eligible for HIV testing in South Africa (Fig 1B).

### Screening questions and HIV testing

In R3, Zambia had 11.3% (3,746/33,283) of eligible participants classified as being “at risk.” Acceptance of HIV testing was 81.3% (3,047/3,746) in the “at risk” and 76.0% (22,453/29,537) in the “not at risk” groups (Fig 1A). In South Africa, 17.5% (1,491/8,504) were classified as “at risk”. Acceptance of HIV testing was 81.4% (1,214/1,491) in the “at risk” and 60.7% (4,255/7,013) in the “not at risk” groups (Fig 1B).

In both countries, there was no substantial difference between the proportions of adolescent boys and girls who were classified “at risk”. In Zambia there was a small increase in the proportion of those who were “at risk” with increase in age, however this pattern was not observed in South Africa. In both countries, there were marked differences between communities. (Table 1A and 1B)

**Table 1:** a. Demographics among all 10–14 year olds screened who did not self-report being HIV-positive (Zambia) and the proportion “at-risk”. b. Demographics among all 10–14 year olds screened, who did not self-report being HIV-positive (South Africa) and the proportion “at-risk”.

	N of all 10–14 year olds eligible for HIV testing	N "at risk" (% among those eligible for testing within each category)	p-value†
**Overall**			
All	33283	3,746/33,283 (11.3%)	
**Sex**			
M	15206	1,716/15,206 (11.3%)	0.874
F	18077	2,030/18,077 (11.2%)	
**Age**			
10	7525	729/7,525 (9.7%)	<0.001
11	6631	723/6,631 (10.9%)	
12	6673	729/6,673 (10.9%)	
13	6396	794/6,396 (12.4%)	
14	6058	771/6,058 (12.7%)	
**Community**			
1	3572	466/3,572 (13.0%)	<0.001
2	1400	106/1,400 (7.6%)	
5	5693	849/5,693 (14.9%)	
6	3267	262/3,267 (8.0%)	
8	6577	606/6,577 (9.2%)	
9	7715	1034/7,715 (13.4%)	
10	2674	223/2,674 (8.3%)	
11	2385	200/2,385 (8.4%)	
	N of all 10–14 year olds eligible for HIV testing	N "at risk" (% among those eligible for testing within each category)	p-value†
**Overall**			
All	8,504	1,491/8,504 (17.5%)	
**Sex**			
M	3,954	689/3,954 (17.4%)	0.808
F	4,550	802/4,550 (17.6%)	
**Age**			
10	1,871	365/1,871 (19.5%)	0.144
11	1,754	291/1,754 (16.6%)	
12	1,680	284/1,680 (16.9%)	
13	1,635	284/1,635 (17.4%)	
14	1,564	267/1,564 (17.1%)	
**Community**			
13	1,577	319/1,577 (20.2%)	<0.001
14	524	59/524 (11.3%)	
16	2,707	484/2,707 (17.9%)	
18	1,501	394/1,501 (26.2%)	
19	917	137/917 (14.9%)	
20	1,278	98/1,278 (7.7%)	

† p-value for association between each demographic factor and being “at risk” from chi-squared test.

In Zambia 122/25,500 (0.5%) of adolescents who tested with CHiPs had a positive result, compared with South Africa where it was 9/5,469 (0.2%). Both Zambia and South Africa had a higher proportion with a positive test result in the “at risk” group compared to the “not at risk.” In Zambia, these proportions were 1.5% (45/3,047) in the “at risk” and 0.3% (77/22,453) among those in the “not at risk” group. In South Africa with fewer newly-diagnosed individuals found, the proportion testing positive was 0.6% (7/1,214) in the “at risk” group and 0.0005% (2/4,253) in the “not at risk” group. (Fig 1A and 1B).

In both countries, there was strong evidence of an association between screening “at risk” and testing positive for HIV. In Zambia those who screened “at risk” were estimated to have 4.6 times the odds of testing HIV-positive compared to those “not at risk”, after adjusting for age, sex and community (95% CI: 3.1–6.6, p<0.001). In South Africa the estimated increase in odds was 16.7 times among those “at risk” (95% CI: 3.4–83.3, p<0.001) (Table 2).

**Table 2:** Association between screening "at risk" and testing HIV positive in the P-ART-Y sub-study of HPTN 071 (PopART).

	Unadjusted			Adjusted*		
	Odds Ratio	95% CI	p-value	Odds Ratio	95% CI	p-value
Zambia	4.4	(3.0–6.3)	<0.001	4.6	(3.1–6.6)	<0.001
South Africa	12.3	(2.6–59.4)	0.002	16.7	(3.4–83.3)	<0.001

* Odds ratio adjusted for age, sex, and community.

### Sensitivity, specificity and predictive values of the Screening tool

The sensitivity, specificity and the positive and negative predictive values were estimated among those who accepted an HIV test from the CHiPs (25,500 in Zambia; and 5,469 in South Africa) (Table 3A and 3B).

**Table 3:** a. Sensitivity and specificity, positive predictive value (PPV), negative predictive value (NPV), number needed to test (NNT) to identify 1 HIV-infected after application of screening tool (Zambia) in the P-ART-Y sub-study of HPTN 071 (PopART). b. Sensitivity and specificity, positive predictive value (PPV), negative predictive value (NPV), number needed to test (NNT) to identify 1 HIV-infected after application of screening tool (South Africa) in the P-ART-Y sub-study of HPTN 071 (PopART).

		Sensitivity (%)	Specificity (%)	PPV (%)	NPV (%)	NNT
	Screened "at risk"	35.3 (27.3, 44.2)^†^	88.9 (88.5, 89.2)^†^	1.5 (1.1, 2.0)	99.7 (99.6, 99.7)	68 (51, 91)
**Individual Questions**						
	Admitted to hospital	5.5 (2.6, 11.2)	96.6 (96.4, 96.9)	0.8 (0.4, 1.6)	99.5 (99.4, 99.6)	130 (62, 273)
	Skin problems	12.6 (7.8, 19.7)	99.1 (99.0, 99.2)	6.1 (3.8, 9.8)	99.6 (99.5, 99.7)	16 (10, 27)
	Parent died	24.3 (17.5, 32.7)	92.8 (92.5, 93.1)	1.6 (1.1, 2.2)	99.6 (99.5, 99.7)	64 (45, 90)
	Poor health	12.6 (7.8, 19.7)	99.0 (98.8, 99.1)	5.5 (3.4, 8.8)	99.6 (99.5, 99.7)	18 (11, 30)
		**Sensitivity (%)**	**Specificity (%)**	**PPV (%)**	**NPV (%)**	**NNT**
	Screened "at risk"	72.3 (26.8, 94.9)^†^	82.5 (81.6, 83.4)^†^	0.6 (0.3, 1.2)	100.0 (99.8, 100.0)	173 (83, 364)
**Individual Questions**						
	Admitted to hospital	20.7 (3.5, 65.0)	93.5 (92.9, 94.1)	0.4 (0.1, 1.8)	99.9 (99.7, 99.9)	224 (56, 900)
	Skin problems	31.0 (7.3, 71.8)	93.9 (93.3, 94.5)	0.7 (0.2, 2.2)	99.9 (99.8, 100.0)	141 (46, 439)
	Parent died	20.7 (3.5, 65.0)	93.3 (92.7, 93.9)	0.4 (0.1, 1.7)	99.9 (99.7, 99.9)	232 (58, 930)
	Poor health*	…	97.5 (97.1, 97.8)	…	99.9 (99.7, 99.9)	

* In SA, among those who tested HIV-positive none answered yes to the question “has the child had poor health in the past 3 months”.

† Sensitivity and specificity are adjusted using inverse probability weighting to account for different testing uptake in at risk and not at risk groups. Unweighted estimates for sensitivity and specificity are:- Zambia sensitivity: 36.9 (28.7–45.9); Zambia specificity: 88.2 (87.8–88.6); SA sensitivity: 77.8 (35.5–95.7); SA specificity: 77.9 (76.8–79.0).

Definitions:

• Positive Predictive Value (PPV) is the probability that subjects with a positive screening test are truly HIV-positive.

• Negative Predictive Value (NPV) is the probability that subjects with a negative screening test truly don’t have the disease.

• Number Needed to Test (NNT) is the number needed to test in order to obtain one positive result

• Sensitivity is how accurate the screening test is in identifying disease in people who truly have the disease.

• Specificity is the accuracy of the screening test in correctly classifying truly non-diseased people.

In Zambia, the screening tool had an estimated sensitivity of 35.3% (95% CI: 27.3%-44.2%) and an estimated specificity of 88.9% (95% CI: 88.5%-89.2%). This resulted in an estimated positive predictive value (PPV) of 1.5% (95% CI: 1.1%-2.0%), giving a number needed to test (NNT) to obtain, on average, one HIV-positive test result of 68 (95% CI: 51–91). In South Africa, the estimated sensitivity was higher at 72.3% (95% CI: 26.8%-94.9%), but this estimate was very imprecise given the very low numbers of undiagnosed HIV-positive 10–14-year-olds. The specificity was estimated to be 82.5% (95% CI: 81.6%-83.4%) and the PPV was 0.6% (95% CI: 0.3%-1.2%) resulting in an estimated NNT of 173 (95% CI: 83–364).

The estimated proportion who were HIV-positive among those who did not self-report HIV-positive was 0.5% (95% CI: 0.4%-0.6%) in Zambia and 0.1% (95% CI: 0.1%-0.3%) in South Africa. This gives a NNT for universal HIV testing in Zambia of 213 (95% CI: 178–254) and 715 (95% CI: 370–1,382) in South Africa.

### Screening questions

The individual questions were investigated to identify if all were appropriate screening questions in this setting. In Zambia, ≈1% answered yes to whether they had recurring skin problems or poor health in the last three months. About 3.5% had ever been admitted to hospital and ≈7% had a parent who had died. In South Africa the prevalence of ever being admitted to hospital (6%) and having skin problems (6%) was higher than in Zambia. Of the four questions, death of a parent was the most sensitive question for testing positive for HIV, with a sensitivity of over 20% in both countries. Previous hospital admission was the least predictive element. (Table 3).

## Discussion

This study explored the use of a validated four-question screening tool to identify HIV-positive adolescents aged 10–14 in community settings in Zambia and South Africa. The study took advantage of the large community intervention that was implemented in the HPTN071 (PopART) trial. The sensitivity of the screening tool was only about 35% in Zambia and somewhat higher in South Africa at 72% (although with a wide confidence interval). These findings are broadly similar to what was found in community settings in Zimbabwe where the sensitivity of the screening tool was 56.3% (95% CI: 44.0–68.1%) [17], and much lower than when applied in clinical settings [10,16].

So by screening using this tool in Zambia it was estimated that by testing just over a tenth of all adolescents, would identify about a third of the HIV-positive cases. In South Africa our best estimate is that we would need to test about a fifth of adolescents in order to identify nearly three-quarters of HIV-positive cases testing, but these estimates are very imprecise given the very low prevalence of undiagnosed HIV in this population.

Since the screening tool was easy to use in both our study and that of Bandason et al. [10,17] it could be an acceptable alternative to universal testing, if there are insufficient resources to test all 10–14 year olds. The screening tool was initially designed to identify children and adolescents infected through mother-to-child-transmission presenting at health facilities. Therefore, the PPV of this tool is likely to drop over time with the move to universal treatment for people living with HIV and improved strategies for prevention-of-mother-to-child-transmission [10,16]. A tool with a higher sensitivity would be desirable for community screening [17]. The value of testing HIV negative is that it provides a pathway for these adolescents in accessing HIV prevention services [24].

A systematic review highlighted that innovative population-based HTC strategies that could easily be brought to scale were needed that could be implemented effectively, efficiently and economically at a population level [11,24]. Similarly WHO cited the need for research in pre-HIV testing screening questions to identify at risk populations [25]. Our study found that this screening tool, when used in a community setting, had a low sensitivity, missing two-thirds of the ALHIV in our population. It did however reduce the number of tests to be performed by around 90% compared with universal testing, so there would be a reduced financial cost associated with performing this pre-screening but a great cost in terms of missed diagnoses and therefore the tool should not be a replacement for universal testing of adolescents.

The strengths of this study included the very large number of young adolescent study participants; use of the community setting for a clinical tool; conducting the study in two countries with a high HIV prevalence; and a study population of adolescents aged 10–14, a sub-population that has never been studied at this scale. The screening tool was simple and was easily administered by community health-care workers.

The limitations are that the study was designed to focus more on finding and testing adolescents who were classified as being “at risk” compared to the “not at risk”, hence the difference in the uptake of HIV testing between these groups especially in South Africa. So to generalise this result it is necessary to make the assumption that those who did not accept a test have the same prevalence of HIV as those who did, within each of the “at risk” or “not at risk” strata.

## Conclusions

The sensitivity of the tool was low in Zambia, with only a third of HIV-positive adolescents identified. Therefore, if this screening tool were applied, two thirds of adolescents with undiagnosed HIV would be missed compared to universal testing. In South Africa the sensitivity estimate was higher, but measured very imprecisely. The screening tool may be of some value where UTT is not possible and limited resources must be prioritised toward adolescents who are more likely to be living with HIV, and may be of greater value if the prevalence of undiagnosed HIV is higher. However, given our goal is to identify and treat all ALHIV, as well as link all HIV uninfected young people to prevention services, this screening tool should not be a substitute for UTT in community settings.

## Supporting information

S1 Data (XLSX)
